# A Novel Member of *Chitinophagaceae* Isolated from a Human Peritoneal Tumor

**DOI:** 10.1128/genomeA.01297-15

**Published:** 2015-11-12

**Authors:** Alexander S. Lo, D. Scott Merrell, Haiyan Lei, Armando Sardi, Thomas McAvoy, Traci L. Testerman

**Affiliations:** a Uniformed Services University, Bethesda, Maryland, USA; b Human Tissue Microbiology Laboratory, Division of Cellular and Gene Therapies, Center for Biologics Evaluation and Research, FDA, Silver Spring, Maryland, USA; c Mercy Medical Center, Baltimore, Maryland, USA; d University of Maryland, College Park, Maryland, USA; e University of South Carolina School of Medicine, Columbia, South Carolina, USA

## Abstract

Peritoneal tumors from a rare malignancy, pseudomyxoma peritonei, frequently contain bacteria. Evidence suggests that tumor-associated bacteria contribute to pseudomyxoma peritonei development and/or progression. One unique isolate (PMP191F) was characterized via whole-genome sequencing using the Illumina MiSeq platform. PMP191F shows similarities to the *Chitinophaga, Niastella*, and *Flavitalea* genera.

## GENOME ANNOUNCEMENT

Pseudomyxoma peritonei (PMP) is a rare malignancy of the peritoneum believed to originate from a mucinous appendiceal cyst adenoma, which invades the intraperitoneal cavity (1). Previously, the presence of enteric bacteria, including *Helicobacter pylori*, has been identified in PMP (2). Additionally, Gilbreath et al. (3) identified a core microbiome present in PMP tumors that is conserved among examined patients. Moreover, a recent study by Semino-Mora et al. (4) suggested higher bacterial density and higher expression of the MUC2 mucin associated with more malignant forms of PMP. It was further shown that the treatment of patients with antibiotics targeting *H. pylori* prior to cytoreductive surgery reduces the peritoneal bacterial density and decreases nuclear β-catenin, a known biomarker of carcinogenesis. Additionally, 5-year survival data suggest increased survivability among antibiotic-treated patients (3). To further understand the relationship between bacteria and PMP, we have sought to isolate, identify, and characterize some of the bacterial species found in PMP samples. Isolate PMP191F showed unique characteristics compared with other PMP isolates and was further characterized.

Bacteria from PMP samples collected under an institutional review board (IRB)-approved protocol were initially grown in Ham’s F-12 medium supplemented with 2% fetal bovine serum. Bacteria were subsequently isolated as single colonies on blood agar plates. Genomic DNA was extracted from the isolates for initial PCR amplification of the 16S rRNA gene. The 16S sequence of PMP191F was found to have 95% identity with an unsequenced *Chitinophaga* species and *Flavitalea populi* strain HY-50R (accession no. HM130561.1). PMP191F genomic DNA was subjected to DNA library construction using the Illumina MiSeq platform and sequenced with a 2.250-bp paired-end sequencing mode. A total of 17.8 million quality reads of 225 bp average length were assembled into 66 contigs (*N*_50_, 269 kbp) using the CLC Genomics Workbench (version 6.0; CLC bio). These contigs yielded a total genome size of 6,452,579 bp and a G+C content of 43.4%. Functional annotation was performed with Rapid Annotations using Subsystems Technology (RAST) (5), which predicted 5,577 coding regions with 380 subsystems and 46 tRNAs. Several genes were predicted to impart antibiotic resistance, including β-lactamase and multiple efflux pumps. *Chitinophaga pinensis* was identified as the closest neighbor, with a RAST score of 532. Comparative analysis of the genome contents using progressiveMauve (6) of previously identified close species estimated 90.3% homology with *C. pinensis* (accession no. NC_013132.1) and 84.7% with *Niastella koreensis* (accession no. CP003178.1); however, 16S sequence-based phylogeny places PMP191F closer to *Niastella* than to *Chitinophaga*. For this reason, it is unclear whether PMP191F belongs to any of the named genera.

Phenotypic, biochemical, and metabolic characterizations were performed using the Biolog ID system (Biolog, Inc., Hayward, CA, USA). Phenotypic analysis using a Biolog assay suggested a relationship to *Flavobacterium johnsoniae*, with a probability and similarity of 0.59. The Biolog assay also revealed resistance to rifamycin and lincomycin.

Given the association between peritoneal bacterial load and inflammatory markers, as well as the improved survival in antibiotic-treated PMP patients (2), we propose PMP191F as a novel bacterial species that has potentially significant associations with PMP.

### Nucleotide sequence accession numbers.

This whole-genome shotgun project has been deposited at DDBJ/EMBL/GenBank under the accession no. LGYU00000000. The version described in this paper is the first version, LGYU01000000.
